# ATP2B3 Inhibition Alleviates Erastin–Induced Ferroptosis in HT-22 Cells through the P62–KEAP1–NRF2–HO-1 Pathway

**DOI:** 10.3390/ijms24119199

**Published:** 2023-05-24

**Authors:** Shihui Guo, Aiying Zhong, Dongxu Zhang, Jiang Gao, Yingdong Ni, Ruqian Zhao, Wenqiang Ma

**Affiliations:** 1 Key Laboratory of Animal Physiology and Biochemistry, Ministry of Agriculture and Rural Affairs, College of Veterinary Medicine, Nanjing Agricultural University, Nanjing 210095, China; 2017107012@njau.edu.cn (S.G.); zhongaiying@stu.njau.edu.cn (A.Z.); 2022107030@stu.njau.edu.cn (D.Z.); 2022007052@stu.njau.edu.cn (J.G.); niyingdong@njau.edu.cn (Y.N.); zhaoruqian@njau.edu.cn (R.Z.); 2 MOE Joint International Research Laboratory of Animal Health & Food Safety, Nanjing Agricultural University, Nanjing 210095, China

**Keywords:** ATP2B3, ferroptosis, HT-22, P62–KEAP1–NRF2, HO-1

## Abstract

Ferroptosis participates in the occurrence and development of neurological disorders. Modulating ferroptosis may have therapeutic potential in nervous system diseases. Therefore, TMTbased proteomic analysis in HT-22 cells was performed to identify erastin–induced differentially expressed proteins. The calcium-transporting ATP2B3 (ATP2B3) was screened as a target protein. ATP2B3 knockdown markedly alleviated the erastin–induced decrease in cell viability and elevated ROS (*p* < 0.01) and reversed the up-regulation of oxidative stress-related proteins polyubiquitin-binding protein p62 (P62), nuclear factor erythroid 2-related factor2 (NRF2), heme oxygenase-1 (HO-1), and NAD(P)H quinone oxidoreductase-1 (NQO1) protein expression (*p* < 0.05 or *p* < 0.01) and the down−regulation of Kelch-like ECH-associated protein 1(KEAP1) protein expression (*p* < 0.01). Moreover, NRF2 knockdown, P62 inhibition, or KEAP1 overexpression rescued the erastin–induced decrease in cell viability (*p* < 0.05) and increase in ROS production (*p* < 0.01) in HT-22 cells, while simultaneous overexpression of NRF2 and P62 and knockdown of KEAP1 partially offset the relief effect of ATP2B3 inhibition. In addition, knockdown of ATP2B3, NRF2, and P62 and overexpression of KEAP1 significantly down-regulated erastin–induced high expression of the HO-1 protein, while HO-1 overexpression reversed the alleviating effects of ATP2B3 inhibition on the erastin–induced decrease in cell viability (*p* < 0.01) and increase in ROS production (*p* < 0.01) in HT-22 cells. Taken together, ATP2B3 inhibition mediates the alleviation of erastin–induced ferroptosis in HT-22 cells through the P62–KEAP1–NRF2–HO-1 pathway.

## 1. Introduction

Neurological diseases are a significant cause of disability in daily life and the second leading cause of death worldwide [1]. It has been shown that cell death and shrinkage in a particular region of the brain are fundamental pathological features of neurodegenerative diseases [2,3,4]. A growing body of evidence indicates ferroptosis is associated with neurodegenerative diseases such as Parkinson’s disease (PD), Alzheimer’s disease (AD), and Huntington’s disease (HD) [5,6,7]. The features of ferroptosis, such as decreased glutathione peroxidase type 4 (GPX4) activity, iron, and lipid peroxide accumulation, are important pathological events in neurodegenerative disorders [8,9,10]. The role of ferroptosis in relieving the symptoms of various neurological disorders is well established. Metal chelators, such as deferiprone (DFP) and deferoxamine (DFO), can tightly bind iron (III) to impede the progression of PD, AD, and secondary injury following intracerebral hemorrhage (ICH), resulting in a notable neuroprotective effect [11,12,13]. Hambright et al. reported that the cerebral cortex and hippocampal neurons of mice lacking GPX4 showed obvious cognitive impairments, whereas the neurodegeneration was alleviated when mice were administered the ferroptosis inhibitor liproxstatin-1 [10]. Moreover, dysregulation of the NRF2-induced ferroptosis occurs in AD [14], while natural products or small molecules activate NRF2 to play a beneficial role in AD [15,16,17]. Therefore, targeting ferroptosis has become a hot research topic and leads to new therapeutics and neurological disease prevention measures.

Ferroptosis is a nonapoptotic form of cell death that occurs with iron deficiency [18]. The ferroptosis process is a complex set of biological pathways that involves three main pathways: glutathione/glutathione peroxidase 4 (GSH/GPX4), iron metabolism, and lipid metabolism pathways [9]. GSH is utilized by GPX4 for the conversion of phospholipid hydroperoxides into lipid alcohols, an action that inhibits ferroptosis [18], while Gpx4 inactivation or depletion could induce ferroptotic cell death [19,20]. Excess free iron triggers reactive oxygen species (ROS) generation via the Fenton reaction, resulting in cell death [21]. In Neuro2a cells, ferritin overexpression significantly reduces ROS levels, induces GPX4 expression, and enhances cell viability [22]. The HO-1 catalyzes the degradation of heme iron chelate as ferrous iron, while enhanced HO-1 expression could induce ferroptosis by promoting free iron accumulation and ROS production [23]. In addition, it is well established that lipid peroxidation (LPO) drives the initiation and execution of ferroptosis [24]. The acyl-CoA synthetase long-chain family 4 (ACSL4) is capable of catalyzing fatty acids to synthesize acyl-CoAs, promote 5-hydroxyeicosatetraenoic acid (5-HETE) production, induce lipid accumulation, and result in ferroptosis [25,26]. It is therefore critical to identify novel therapeutic targets, predictive targets, and regulatory targets for the prevention and treatment of ferroptosis by studying proteins modulating ferroptosis.

It has been found that erastin targets mitochondrial networks, inhibiting mitochondrial voltage-dependent anion channels and P53, leading to the accumulation of ROS and cancer cell death without apoptosis [27,28,29]. Erastin has been used to develop a ferroptosis model in SH-SY5Y neuroblastoma cells, tumor cells, and HT-22 cells [30,31,32]. However, the role of ferroptosis in erastin–induced neuronal toxicity is not well understood. In the current study, calcium-transporting ATPase (ATP2B3) was screened in erastin–induced HT-22 cells via TMT (tandem mass tag) quantitative proteomics. We provide evidence that ATP2B3 regulates ROS production to inhibit erastin–induced ferroptosis sensitivity. Mechanically, ATP2B3 knockdown alleviates ferroptosis in HT-22 cells under erastin treatment via the P62–KEAP1–NRF2–HO-1 pathway. Our findings present a novel role for ATP2B3 in the regulation of oxidative, iron-independent ferroptosis and provide a new therapeutic target to prevent neuronal cells from developing ferroptosis.

## 2. Results

### 2.1. Erastin Down–Regulates ATP2B3 Protein Level in HT-22 Cells

**Figure 1:**
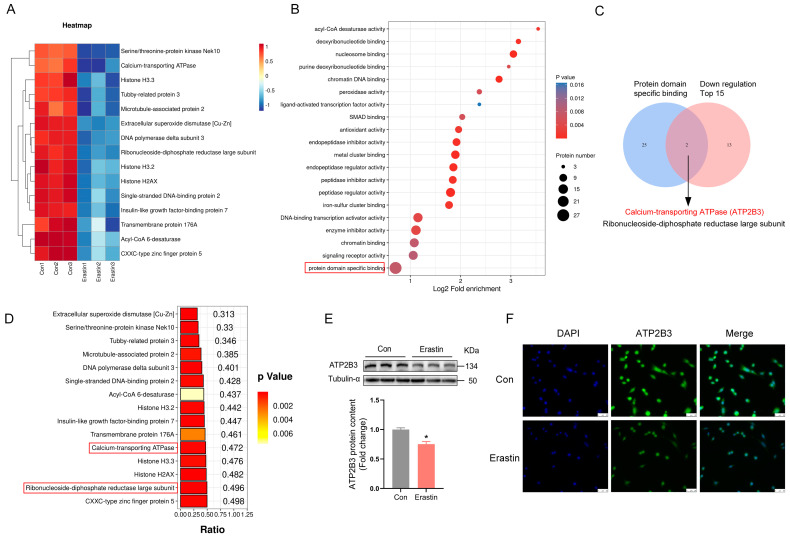
ATP2B3 is down−regulated in erastin−treated HT-22 cells. (**A**) Cluster heat map of Top15 down-regulated differential proteins; (**B**) Top 20 pathways for down-regulated differential proteins based on GO-molecular function enrichment analysis, the pathways with the highest enrichment of differential proteins are shown in red box; (**C**) ATP2B3 and RRMI at the intersection of Top15 down-regulated differential proteins and “protein domain specific binding” pathway-associated proteins; (**D**) Histograms of *p*-Ratio values of Top15 down-regulated differential proteins, the red box shows the protein in the intersection; (**E**,**F**) Western blot and immunofluorescence were used to detect the protein content of ATP2B3. DAPI was used for nuclear staining, and the cells were photographed and analyzed by fluorescence microscope, scale bar: 50 μm. Con: control, n = 3. Values are means ± SEM, * *p* < 0.05.

To gain a comprehensive, unbiased overview of cellular protein dynamics, we investigated proteomic changes in erastin–induced HT-22 cells by conducting a TMT–based proteomics analysis. As demonstrated in Figure 1A, the Top 15 decreased proteins were displayed in HT-22 cells treated with erastin. GO–Molecular Function analysis of the total of 411 markedly down–regulated proteins revealed the strongest links to the pathway “protein domain specific binding” (Figure 1B). The intersection of the Top15 down–regulated proteins and “protein domain specific binding” pathway-associated proteins was taken to screen out the two proteins: calcium-transporting ATP2B3 and ribonucleoside-diphosphate reductase large subunit (RRMI) (Figure 1C). However, ATP2B3 exhibited a larger difference in expression abundance compared to RRMI, according to the *p*-Raito histogram (Figure 1D). Meanwhile, western blot (*p* < 0.05, Figure 1E) and immunofluorescence assay (Figure 1F) confirmed that erastin reduced ATP2B3 expression in HT-22 cells, which was consistent with proteomics. Therefore, ATP2B3 was selected as a target protein to explore its role in the ferroptosis of HT-22 cells under erastin treatment.

### 2.2. ATP2B3 Knockdown Alleviates Ferroptosis in Erastin–Induced HT-22 Cells

**Figure 2:**
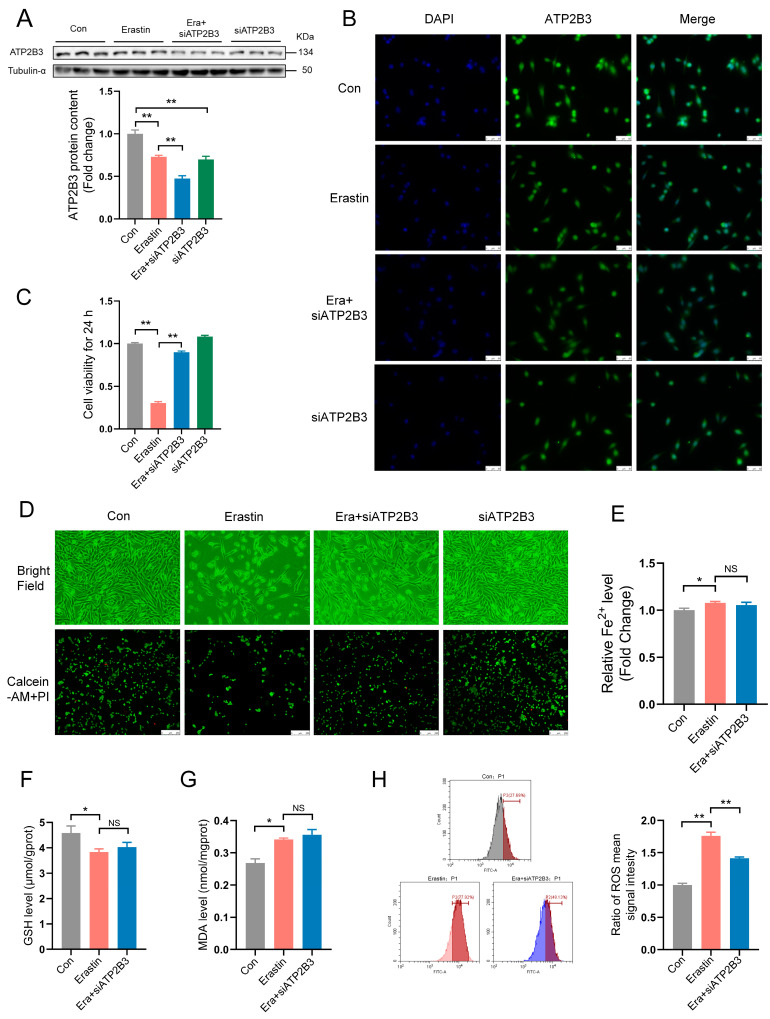
ATP2B3 knockdown alleviates erastin–induced HT-22 ferroptosis. (**A**,**B**) Western blot and immunofluorescence were used to detect the ATP2B3 protein content. DAPI was used for nuclear staining. Morphological observation with staining cells by fluorescence microscope (n = 3), scale bar: 50 μm; (**C**) Cell viability was assayed by CCK-8 kit (n = 4); (**D**) Representative cell morphological changes are shown (up). Calcein-AM staining (green) represents live cells while PI staining (red) represents dead cells (down), scale bar: 250 μm; (**E**) Iron assay kit was used to analyze the intracellular Fe^2+^ content (n = 3); (**F**,**G**) Intracellular GSH/MDA level was measured by GSH/MDA assay kits (n = 3); (**H**) Intracellular ROS levels were detected using flow cytometry (n = 3). Con: control, Era + siATP2B3: siATP2B3 was pre-transfected for 12 h before erastin treatment. Values are means ± SEM, * *p* < 0.05, ** *p* < 0.01, and “NS” indicates no significant difference.

Western blot (*p* < 0.01, Figure 2A) and immunofluorescence (Figure 2B) showed that transfection with siATP2B3 further enhanced erastin–induced ATP2B3 protein reduction in HT-22 cells. Pre−transfection of siATP2B3 followed by erastin treatment significantly mitigated the erastin–induced decline in cell viability (*p* < 0.01, Figure 2C) and reduction in the number of living cells (Figure 2D). In addition, siATP2B3 transfection also impaired erastin–induced elevation of ROS levels in HT-22 cells (*p* < 0.01, Figure 2H). However, the erastin–induced changes in Fe^2+^ content and MDA level, as well as GSH level, could not be rescued in HT-22 cells transfected by siATP2B3 (*p* > 0.05, Figure 2E–G). These results indicated that ATP2B3 inhibition alleviated ferroptosis in erastin–induced HT-22 cells by reducing ROS production.

### 2.3. ATP2B3 Inhibition Regulates Oxidative Stress-Related Genes and Proteins in Erastin–Induced HT-22 Cells

**Figure 3:**
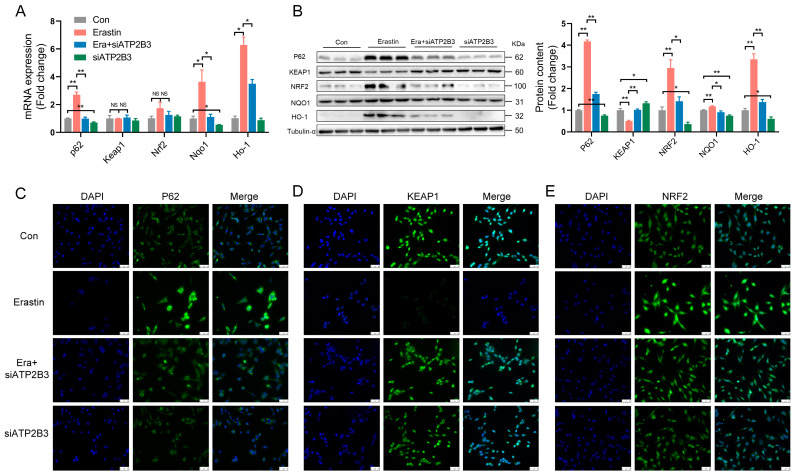
ATP2B3 inhibition reverses oxidative stress−related proteins’ expression changes in erastin–induced HT-22 cells. (**A**) RT-PCR analysis of *p62*, *Keap1*, *Nrf2*, *Nqo1* and *Ho-1* genes expression; (**B**) Western blot analysis of P62, KEAP1, NRF2, NQO1 and HO-1 protein levels; (**C**–**E**) Immunofluorescence staining of P62, KEAP1 or NRF2 (green). DAPI was used for nuclear staining (blue). Morphological observation with staining cells by fluorescence microscope, scale bar: 50 μm. Con: control, Era + siATP2B3: siATP2B3 was pre-transfected for 12 h before erastin treatment. n = 3, Values are means ± SEM, * *p* < 0.05, ** *p* < 0.01, “NS” indicates no significant difference.

The P62−KEAP1−NRF2 pathway participates in ferroptosis by affecting intracellular oxidative stress [33]. ATP2B3 inhibition delays the erastin–induced increase in mRNA levels of p62, Nqo1, and Ho-1 in HT-22 cells (*p* < 0.01 or *p* < 0.05, Figure 3A). Erastin markedly increased the protein expression of P62, NRF2, and HO-1 and decreased KEAP1 protein expression in HT-22 cells. siATP2B3 transfection also reversed the erastin–induced expression changes of oxidative stress-related proteins, including P62, NRF2, HO-1, and KEAP1, in HT-22 cells (*p* < 0.01 or *p* < 0.05, Figure 3B). Immunofluorescence results demonstrated that ATP2B3 inhibition delayed the up-regulation of NRF2 and P62 proteins, as well as the down-regulation of KEAP1 protein in erastin−treated HT-22 cells (Figure 3C–E).

### 2.4. ATP2B3 Inhibition Mitigates Erastin–Induced HT-22 Ferroptosis via Reducing P62 Protein Expression

**Figure 4:**
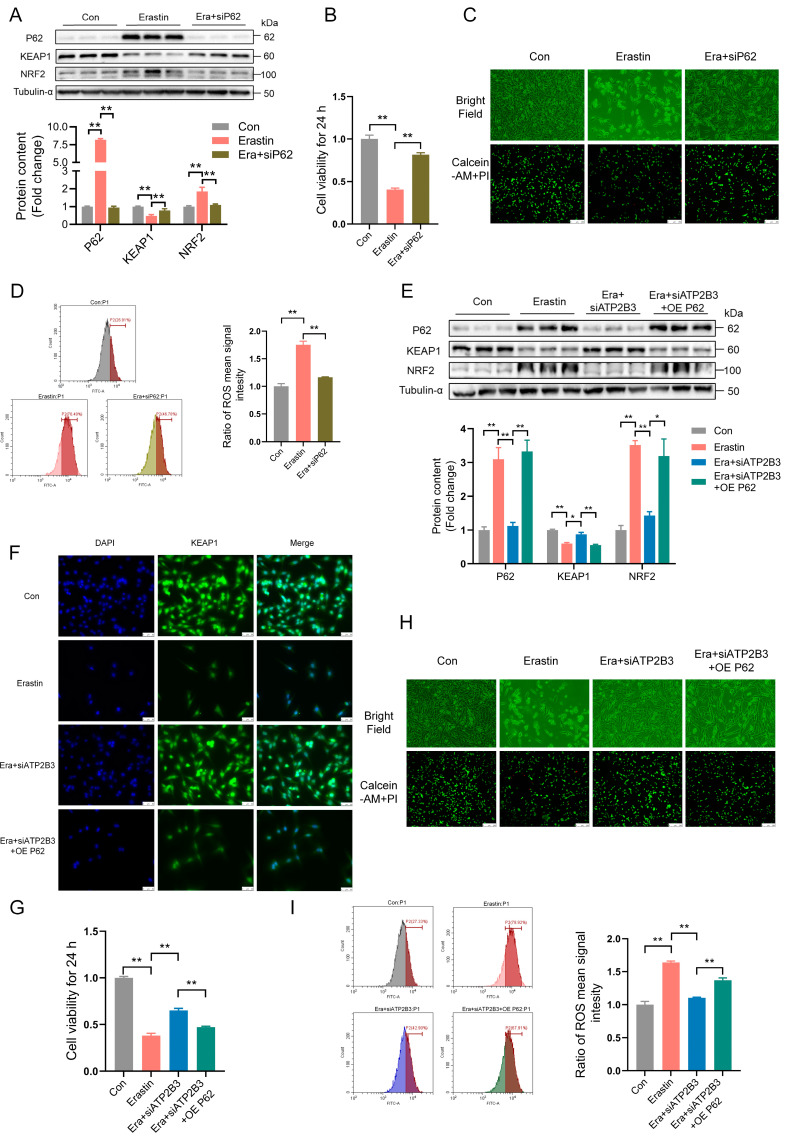
P62-mediated ATP2B3 knockdown relieves erastin–induced HT-22 ferroptosis. (**A**) Western blot was used to detect P62, KEAP1, and NRF2 proteins content (n = 3); (**B**) Cell viability was assayed by CCK-8 kit (n = 4); (**C**) Representative cell morphological changes are shown (up); Calcein-AM staining (green) represents live cells while PI staining (red) represents dead cells (down), scale bar: 250 μm; (**D**) Intracellular ROS levels were measured using flow cytometry (n = 3); (**E**) Western blot was used to detect the P62, KEAP1, and NRF2 proteins content; (**F**) Immunofluorescence was used to detect the protein content of KEAP1, DAPI was used for nuclear staining, scale bar: 50 μm; (**G**) Cell viability was assayed by CCK-8 kit (n = 4); (**H**) Representative cell morphological changes are shown (up), Calcein-AM staining (green) represents live cells while PI staining (red) represents dead cells (down), scale bar: 250 μm; (**I**) Intracellular ROS levels were measured using flow cytometry (n = 3). Con: control, Era + siATP2B3: siATP2B3 was pre-transfected for 12 h before erastin treatment, Era + siP62: siP62was pre-transfected for 12 h before erastin treatment, Era + siATP2B3 + OE P62: siATP2B3 and OE P62 were pre-transfected for 12 h before erastin treatment. Values are means ± SEM, * *p* < 0.05, ** *p* < 0.01.

The HT-22 cells were pre-transfected with siP62 for 12 h and then treated with erastin for 24 h. siP62 transfection significantly reduced the protein expression of P62 and NRF2, enhanced KEAP1 protein expression (*p* < 0.01, Figure 4A), and greatly alleviated the erastin–induced decrease in cell viability (*p* < 0.05, Figure 4B), reduced the number of living cells (Figure 4C), and increased intracellular ROS levels (*p* < 0.01, Figure 4D) in HT-22 cells. According to the results of western blot and immunofluorescence, overexpression of P62 promoted the increasing P62 and NRF2 protein expression (*p* < 0.01 or *p* < 0.05, Figure 4E) and the decreasing KEAP1 protein expression (*p* < 0.01, Figure 4E,F) in HT-22 cells under erastin and siATP2B3 treatment. The alleviating effects of siATP2B3 on erastin–induced decreased cell viability (*p* < 0.01, Figure 4G), reduced number of living cells (Figure 4H), and increased ROS levels (*p* < 0.01, Figure 4I) were offset by P62 overexpression. These results indicated that the mitigation effect of siATP2B3 on erastin–induced HT-22 ferroptosis was mediated through the reduction of P62 protein expression.

### 2.5. ATP2B3 Inhibition Relieves Erastin–Induced HT-22 Ferroptosis through IncressingKEAP1 Expression

**Figure 5:**
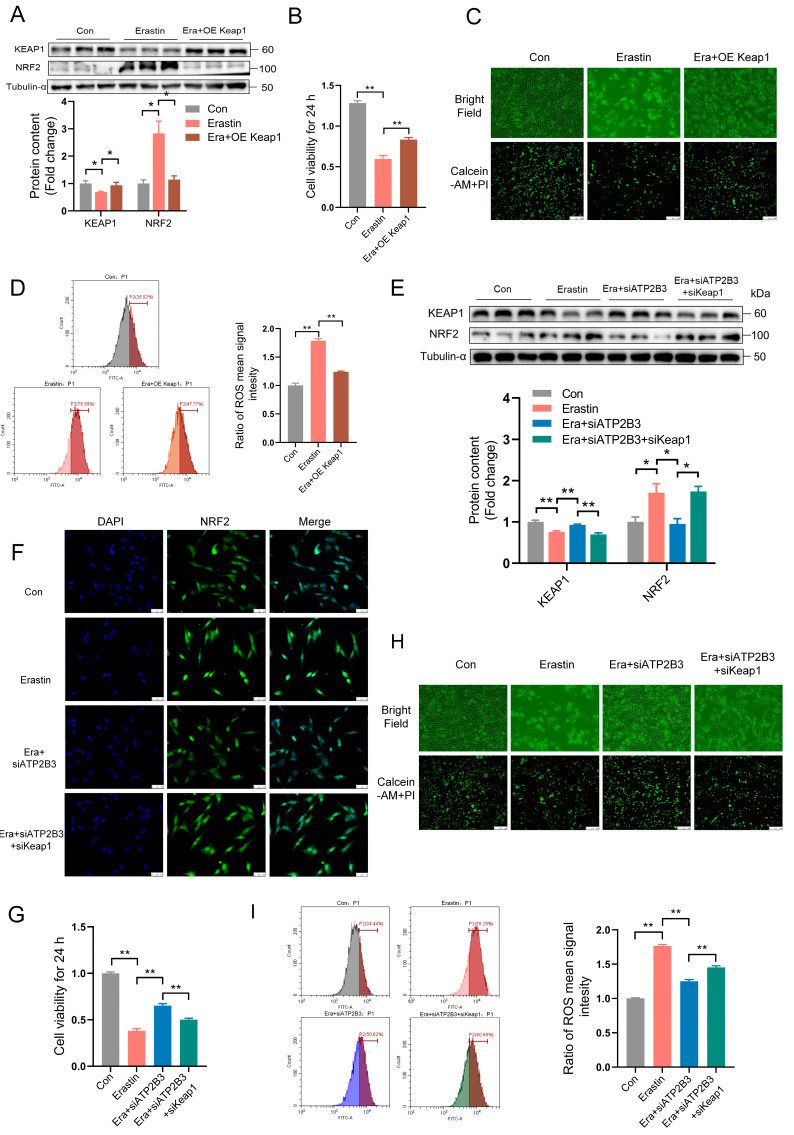
KEAP1−mediated ATP2B3 knockdown alleviates erastin–induced HT-22 ferroptosis. (**A**) Western blot was used to detect KEAP1 and NRF2 proteins content (n = 3); (**B**) Cell viability was assayed by CCK-8 kit (n = 4); (**C**) Representative cell morphological changes are shown (up), Calcein-AM staining (green) represents live cells while PI staining (red) represents dead cells (down), scale bar: 250 μm; (**D**) Intracellular ROS levels were measured using flow cytometry (n = 3); (**E**) Western blot was used to detect the KEAP1 and NRF2 proteins content (n = 3); (**F**) Immunofluorescence was used to detect the protein content of NRF2, DAPI was used for nuclear staining (n = 3), scale bar: 50 μm; (**G**) Cell viability was assayed by CCK-8 kit (n = 4); (**H**) Representative cell morphological changes are shown (up); Calcein-AM staining (green) represents live cells while PI staining (red) represents dead cells (down), scale bar: 250 μm; (**I**) Intracellular ROS levels were measured using flow cytometry (n = 3). Con: control, Era + siATP2B3: siATP2B3 was pre-transfected for 12 h before erastin treatment, Era + OE Keap1: OE Keap1 was pre-transfected for 12 h before erastin treatment, Era + siATP2B3 + siKeap1: siATP2B3 and siKeap1 were pre-transfected for 12 h before erastin treatment. Values are means ± SEM, * *p* < 0.05, ** *p* < 0.01.

Keap1 overexpression markedly increased KEAP1 protein expression and decreased NRF2 protein expression in the HT-22 cells under erastin treatment (*p* < 0.05, Figure 5A). Moreover, Keap1 overexpression significantly alleviated the erastin–induced decrease in cell viability (*p* < 0.01, Figure 5B), reduced the number of living cells (Figure 5C), and ROS increases (*p* < 0.01, Figure 5D). In order to further prove that ATP2B3 participated in the process of ferroptosis through KEAP1, siKeap1 was used to inhibit the expression of KEAP1. Indeed, according to the results of western blot and immunofluorescence, KEAP1 knockdown promoted the decrease of KEAP1 protein level (*p* < 0.01, Figure 5E) and the increase of NRF2 protein level (*p* < 0.05, Figure 5E,F) in HT-22 cells under erastin and siATP2B3 treatment. The alleviating effects of siATP2B3 on erastin–induced decreased cell viability (*p* < 0.01, Figure 5G), reduced number of living cells (Figure 5H), and increased ROS levels (*p* < 0.01, Figure 5I) were rescued by KEAP1 knockdown. These results indicated that the mitigation effect of siATP2B3 on erastin–induced HT-22 ferroptosis was mediated through increasing KEAP1 protein expression.

### 2.6. ATP2B3 Inhibition Alleviates Erastin–Induced HT-22 Ferroptosis through Lowering NRF2 Expression

**Figure 6:**
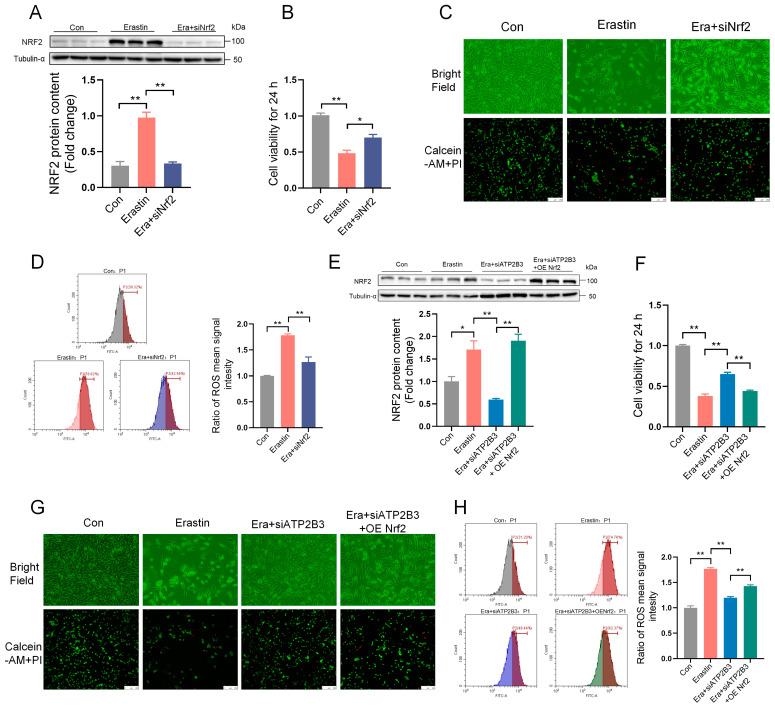
NRF2-mediated ATP2B3 knockdown mitigates erastin–induced HT-22 ferroptosis. (**A**) Western blot was used to detect the NRF2 protein content (n = 3); (**B**) Cell viability was assayed by CCK-8 kit (n = 4); (**C**) Representative cell morphological changes are shown (up); Calcein-AM staining (green) represents live cells while PI staining (red) represents dead cells (down), scale bar: 250 μm; (**D**) Intracellular ROS levels were measured using flow cytometry (n = 3); (**E**) Western blot was used to detect the NRF2 protein content (n = 3); (**F**) Cell viability was assayed by CCK-8 kit (n = 4); (**G**) Representative cell morphological changes are shown (up); Calcein-AM staining (green) represents live cells while PI staining (red) represents dead cells (down), scale bar: 250 μm; (**H**) Intracellular ROS levels were measured using flow cytometry (n = 3). Con: control, Era + siATP2B3: siATP2B3 was pre-transfected for 12 h before erastin treatment, Era + siNrf2: siNrf2 was pre-transfected for 12 h before erastin treatment, Era + siATP2B3 + OE Nrf2: siATP2B3 and OE Nrf2 were pre-transfected for 12 h before erastin treatment. Values are means ± SEM, * *p* < 0.05, ** *p* < 0.01.

siNrf2 was used to inhibit the expression of the NRF2 protein. siNrf2 transfection greatly reduced the protein expression of NRF2 (*p* < 0.01, Figure 6A). NRF2 knockdown alleviated the erastin–induced decrease in cell viability (*p* < 0.05, Figure 6B), reduced the number of living cells (Figure 6C), and ROS increases (*p* < 0.01, Figure 6D) in HT-22 cells. In order to further prove that ATP2B3 participated in the process of ferroptosis through KEAP1, the NRF2 overexpression plasmid (OE Nrf2) was used to enhance the expression of the NRF2 protein. Indeed, NRF2 overexpression promoted NRF2 protein expression (*p* < 0.01, Figure 6E), ROS production (*p* < 0.01, Figure 6H), and the decrease of cell viability (*p* < 0.01, Figure 6F) and number of living cells (Figure 6G) in the HT-22 cells under erastin and siATP2B3 treatment. These results indicated that the mitigation effect of siATP2B3 on erastin–induced HT-22 ferroptosis was achieved by inhibiting NRF2 overactivation.

### 2.7. ATP2B3 Inhibition Mitigates Erastin–Induced HT-22 Ferroptosis through P62−KEAP1−NRF2-HO-1 Pathway

**Figure 7:**
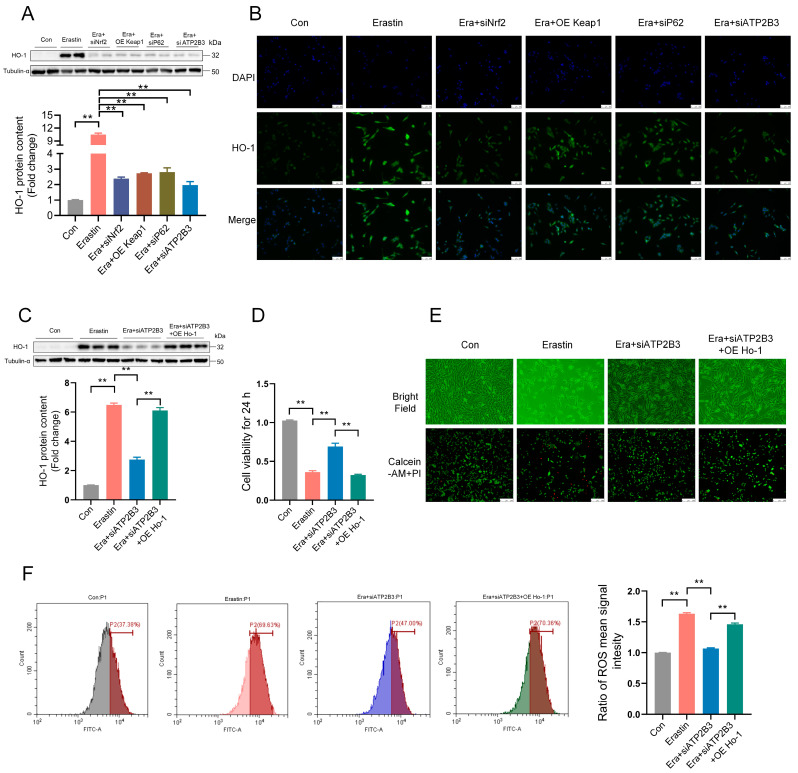
HO-1-mediated ATP2B3 knockdown alleviates erastin–induced HT-22 ferroptosis. (**A**–**C**) Western blot and immunofluorescence were used to detect HO-1 protein content (n = 3), scale bar: 50 μm; (**D**) Cell viability was assayed by CCK-8 kit (n = 4); (**E**) Representative cell morphological changes are shown (up), Calcein-AM staining (green) represents live cells while PI staining (red) represents dead cells (down), scale bar: 250 μm; (**F**) Intracellular ROS levels were measured using flow cytometry (n = 3). Con: control, Era + siATP2B3: siATP2B3 was pre-transfected for 12 h before erastin treatment, Era + siP62: siP62was pre-transfected for 12 h before erastin treatment, Era + siNrf2: siNrf2 was pre-transfected for 12 h before erastin treatment, Era + OE Keap1: OE Keap1 was pre-transfected for 12 h before erastin treatment, Era + siATP2B3 + OE HO-1: siATP2B3 and OE HO-1 were pre-transfected for 12 h before erastin treatment. Values are means ± SEM, ** *p* < 0.01.

HO-1, an Nrf2-target gene, is modulated by the transcriptional factor Nrf2 via binding to the antioxidant response elements (ARE) in the promoter region of the Ho-1 gene [34]. SiNrf2, OE Keap1, siP62, or siATP2B3 markedly inhibited the high level of HO-1 expression in HT-22 cells after erastin treatment (*p* < 0.01, Figure 7A,B). In order to further prove that ATP2B3 participated in the process of ferroptosis through HO-1, the HO-1 overexpression plasmid (OE Ho-1) was constructed and used to enhance the protein level of HO-1 expression. HO-1 overexpression promoted HO-1 protein expression (*p* < 0.01, Figure 7C), ROS production (*p* < 0.01, Figure 7F), as well as the decrease of cell viability (*p* < 0.01, Figure 7D) and number of living cells (Figure 7E) in the HT-22 cells under erastin and siATP2B3 treatment. These results indicated that the mitigation effect of ATP2B3 knockdown on erastin–induced HT-22 ferroptosis was mediated through the P62−KEAP1−NRF2-HO-1 pathway.

## 3. Discussion

Neurological disorders such as stroke, dementia, and traumatic brain injury are associated with ferroptosis, according to emerging evidence. In our study, we found that ATP2B3 inhibition reduced the sensitivity of HT-22 to erastin–induced ferroptosis. ATP2B3 is a protein pump that exports intracellular calcium out of cells, and its mutation can lead to higher basal Ca^2+^ levels in cells by reducing the capacity to export Ca^2+^ [35]. While metabolic disturbance of cellular Ca^2+^ is known to drive ferroptosis through interplay with iron, ROS production, and mitochondrial dysfunction [36,37,38,39]. Unexpectedly, in our study, we found that ATP2B3 knockdown did not affect Fe^2+^ content in erastin–induced HT-22 cells but did alleviate erastin–induced ROS production. ROS overproduction can damage mitochondrial structure and function, ultimately leading to ferroptosis [40]. Therefore, the regulation of ROS production has been shown to play a key role in ATP23-mediated ferroptosis insensitivity.

The P62/KEAP1/NRF2 pathway has been shown to play a key role in oxidative stress and ferroptosis [41,42,43]. In its Keap1-interacting region (KIR), P62 directly binds to the Kelch-repeat domain of Keap1, competing with Keap1 to bind NRF2 [33,44]. Under normal conditions, KEAP1 binds and retains NRF2 in the cytoplasm, preventing NRF2 nuclear translocation [45]. Under oxidative stress, KEAP1 dissociates from NRF2, allowing it to translocate to the nucleus and initiate compensatory gene expression, including HO-1 and NQO1, which participate in ferroptosis [43,46,47]. Furthermore, NRF2 plays an important role in cancer prevention and progression; activation of NRF2/KEAP1 signaling in cancer cells results in chemoresistance, inactivating drug-mediated oxidative stress, and protecting cancer cells from drug-induced cell death [48,49,50]. In the present study, ATP2B3 inhibition down-regulated P62 and NRF2 protein expression and up-regulated KEAP1 protein expression in erastin–induced HT-22 cells. Moreover, P62 overexpression offset ATP2B3 knockdown-mediated expression inhibition of NRF2 and expression improvement of KEAP1 in erastin–induced HT-22 cells. Our rescue analyses further displayed that overexpression of P62 and NRF2, or KEAP1 knockdown, reversed the relief effects of ATP2B3 knockdown on erastin–induced ROS elevation and cell viability decline in HT-22 cells. Therefore, ATP2B3 inhibition triggers ferroptosis insensitivity in erastin–induced HT-22 cells via the P62-KEAP1-NRF2 pathway.

HO-1, NRF2’s principal target, plays a double role in ferroptosis, depending on context as well as cell type. HO-1 acts as a protective factor in mitigating ferroptotic cell death in some cases, while in other cases, it induces ferroptotic cell death. It is reported that HO-1 deficiency displays more sensitivity to erastin–induced ferroptosis in renal proximal tubule cells [51]. The secretion of HO-1 by hepatocellular carcinoma cells treated with erastin has been demonstrated to protect them from ferroptosis [33]. Moreover, the activation of the NRF2/HO-1 pathway attenuated ferroptosis in a para-cetamol-induced liver injury model [52]. In addition, HO-1 overexpression exhibited a more profound effect on ferroptotic induction. In an earlier publication, Kwon et al. reported that HO-1 induced ferroptosis more rapidly in HO-1^+/+^ lung fibroblasts and that a lack of HO-1 attenuated this decrease in cell viability [23]. Luo et al. reported that celastrol could inhibit liver fibrosis by inducing HO-1 overexpression, leading to accumulating ROS production and triggering ferroptosis in hepatic stellate cells [53]. Tagitinin C, Doxorubicin, and Withaferin A could induce NRF2 nuclear translocation and significantly increase the expression level of the HO-1 protein, which promotes lipid peroxidation and induces ferroptosis in cardiomyocytes, cancer cells, or neuroblastoma [54,55,56].

In the current study, increasing expression of HO-1 augmented ferroptosis by accumulating ROS production in HT-22 cells, in agreement with the results in tumor cells [57,58]. In addition, we found that knockdown of ATP2B3, P62, or NRF2 and overexpression of KEAP1 down-regulated the overexpression of HO-1 in HT-22 cells after erastin treatment. In rescue assays, overexpression of HO-1 countervailed the relief effect on ROS increases and cell viability declines caused by ATP2B3 knockdown. Thus, ATP2B3 knockdown exerts neuroprotective effects by reversing the activation of HO-1 in erastin–induced HT-22 cells.

## 4. Materials and Methods

### 4.1. Cell Culture, Transfection, and Treatments

The HT-22 cell line was purchased from Shanghai HuiYing Biological Technology Co., Ltd. (China). An atmosphere containing 5% CO_2_ was used to culture HT-22 cells at 37 °C in 10% fetal bovine serum (FS301-02, TransGen Biotech, Beijing, China), 100 IU/Ml penicillin/streptomycin (FG101-01, TransGen Biotech, Beijing, China), and 90% DMEM (319-005-CL, Wisent, St-Bruno, QC, Canada). An in vitro model of ferroptosis was established by the ferroptosis inducer erastin (S7242, Selleck, Shanghai, China), which was resuspended in DMSO (final concentration was less than 1%). HT-22 cells were transfected with siRNA (50 nM)/overexpressed plasmid (50 nM) (Tsingke, Nanjing, China) with the transfection reagent jetPRIME (114-15, Polyplus Transfection, Illkirch, France) according to the manufacturer’s protocol. Knockdown and upregulation efficiency were measured by Western blot. The siRNA sequences are as follows: siATP2B3:5′-CGATGGTGTGCTCATCCAATT-3′; siP62: 5′-CCACAGGGCTGAAGGAAGCTT-3′; siKeap1:5′-ATATCTACATGCACTTCGGTT-3′; siNrf2: 5′-CCCGAATTACAGTGUCTTAATTT-3′; siHo-1: 5′-AGCCACACAGCACTATGTAAATT-3′.

### 4.2. Cell Viability Assay

In 48-well plates, HT-22 cells were seeded at a density of 1 × 10^4^ cells/well, incubated at 37 °C, and treated with the specified compounds, erastin, with or without transfected siRNA or an overexpressed plasmid. Cell viability was evaluated using the Cell Counting Kit-8 (K1018, ApexBio Technology, Shanghai, China). Briefly, after treatment for 12 or 24 h, 25 μL CCK-8 reagent was added to each well, and the plants were incubated at 37 °C for 1 h. Finally, a full-wavelength multi-function microplate reader (Synergy H1, BioTek, VT, USA) was used to measure OD_450_.

### 4.3. Live/Dead Cell Staining

The treated cells were washed three times with PBS buffer, then incubated with 4.5 mM PI solution and 2 mΜ Calcein-AM (C542, Beijing Tongren Institute of Chemistry, Beijing, China) at 37 °C for 15 min. Cells were photographed under a confocal laser scanning microscope with Z-scan analysis. The Calcein-AM staining of live cells revealed green colors, while the PI staining of dead cells revealed red colors.

### 4.4. Measurement of ROS by Flow Cytometry

In order to detect ROS levels in the cells, 6-well plates were plated with CellROX^®^ Green Reagent (#Cat: C10444, Invitrogen, Carlsbad, CA, USA) at a concentration of 5 μM. Cells were vigorously mixed to adhere to the reagent. A balanced salt solution of Hank’s was used to wash the cells twice after incubation for 30 min at 37 °C. The fluorescence of CellROX^®^ was detected by flow cytometry.

### 4.5. Malondialdehyde (MDA) and Glutathione (GSH) Assay

GSH and MDA content were detected using a GSH assay kit (A061-2-1, JianCheng, Nanjing, China) and an MDA assay kit (A003-1-2, JianCheng, Nanjing, China). Lastly, the absorbance was measured using a microplate reader (Synergy H1, BioTek, Winooski, VT, USA) according to the manufacturer’s instructions.

### 4.6. RNA Isolation and Real-Time PCR

Using TRIzol reagent (15596018, Tsingke Biotechnology Co., Nanjing, China), total RNA was extracted from cultured cells following the methods as previously described [59]. In brief, Trizol was used to lyse the cells after they were washed with PBS. After chloroform was used to isolate RNA, isopropanol was used to precipitate the RNA, which was rinsed twice with ethanol, dried, and resuspended in DEPC water. Furthermore, a cDNA synthesis mix (AU341-02, TransGen Biotech Co., Beijing, China) was used to synthesize the template complementary DNA from 1 μg total RNA. With the Mx3000P Real-Time PCR System, 1 oz of diluted complementary DNA (1:20, *v*:*v*) was used for real-time qPCR (Stratagene, San Diego, CA, USA). All primers (Supplementary Table S1) were synthesized by Tsingke Biotechnology (Nanjing, China). Treatment had no effect on RPL, which was used as a reference gene. An analysis of the data was conducted using 2^−ΔΔCT^.

### 4.7. Protein Extraction and Western Blot Assay

The protein concentrations were measured by the Easy Ⅱ Protein Quantitative Kit (DQ111, TransGen Biotech Co., Beijing, China) according to the manufacturer’s instructions. Ten and thirty grams of proteins were electrophoresed on polyacrylamide gels containing 10% and 12% sodium dodecyl sulfate and transferred to PVDF membranes. Subsequently, the images were captured using the VersaDoc 4000MP system (Bio-Rad, Hercules, CA, USA), and band density was measured using Quantity One (Bio-Rad, Hercules, CA, USA). A tubulin control was used as an internal control. The information on primary and secondary antibodies is described in Supplementary Table S2.

### 4.8. Quantitative Proteomics Analysis

As part of TMT proteomics analysis, proteins were prepared, digested by trypsin, performed using TMT labels, conducted by HPLC fractionation, and carried out by LC-MS/MS analysis. Detailed procedures were described in the Supplementary methods. TMT proteomics analysis and GO analysis in our research were supported by Hangzhou Lianchuan Biotechnology Co., Ltd.

### 4.9. Immunofluorescence (IF) Assays

Immunofluorescence staining was performed according to the previously published method [60]. The detection and analysis of endogenous and transfected proteins was performed using immunofluorescence assays. In brief, cells were grown on slides, fixed with 4% paraformaldehyde for 20 min, treated with 0.5% TritonX-100 for 20 min, blocked with 5% bovine serum albumin for 1 h, and stained with primary antibodies, respectively. The slides were then washed, incubated with Alexa Fluor^®^ 488 Goat Anti-Rabbit IgG (H + L), and the nuclei was stained with 4′, 6-diamidino-2-phenylindole (DAPI, Vector). Using a fluorescence microscope (DMI6000 B, Leica Microsystems, Wetzlar, Germany), mounted slides were observed. The primary antibodies are listed in Supplementary Table S2.

### 4.10. Fe^2+^ Assay

Following the operating manual, we used the iron assay kit (ab83366, Abcam, UK) to detect divalent Fe^2+^ content in HT-22 cells. Briefly, samples were omogenized in iron assay buffer on ice to collect the supernatant. Incubation was carried out for 60 min at 37 °C with the iron probe added to the supernatant. Optical density was immediately detected using a colorimetric microplate reader at 593 nm (Synergy H1, BioTek, VT, USA).

### 4.11. Statistical Analysis

Each biological repeat was performed at least three times and presented means ± standard error of the mean (SEM). Prism 8 software program (GraphPad Software, La Jolla, CA, USA) was used to compare the means for one-way analysis of variance (multiple groups) or t-tests (two groups). The differences were considered statistically significant when *p* < 0.05.

## 5. Conclusions

Taken together, our study provides evidence that ATP2B3 knockdown induces ferroptosis insensitivity in erastin−treated HT-22 cells through a reduction in ROS production. We also demonstrate the inhibitory effect of ATP2B3 knockdown on the P62−KEAP1−NRF2−HO-1 signaling pathway, which has potential implications for the treatment of neurological diseases. Future studies should focus on confirming the neuroprotective effects of ATP2B3 in vivo.

## Data Availability

Not applicable.

## Supplementary Materials

The supporting information can be downloaded at: https://www.mdpi.com/article/10.3390/ijms24119199/s1.
