# Supplementary material for: ATP2B3 Inhibition Alleviates Erastin–Induced Ferroptosis in HT-22 Cells through the P62–KEAP1–NRF2–HO-1 Pathway
Source: Int J Mol Sci. 2023 May 24;24(11):9199. doi: 10.3390/ijms24119199 (PMC10252295; doi:10.3390/ijms24119199)
Supplement: Supplementary file 1 [file ijms-24-09199-s001.zip › ijms-2398751-supplementary.pdf]

Supplementary Table S1. Nucleotide sequences of specific primers

Target genes	Primer sequences (5'to 3')	GenBank No.
Gpx4	F: TACAGGGGTTTCGTGTGCAT<br>R: ACCACACTCAGCATATCGGG	NM_001037741.4
Ptgs2	F: CATCCCCTTCCTGCGAAGTT<br>R: CATGGGAGTTGGGCAGTCAT	NM_011198.5
Srxn1	F: TACCAATCGCCGTGCTCATC<br>R: AGGTCTGAAAGGGTGGACCT	NM_029688.5
Nqo1	F: CATTGCAGTGGTTTGGGGTG<br>R: TCTGGAAAGGACCGTTGTCG	NM_008706.5
Ho-1	F: GAAATCATCCCTTGCACGCC<br>R: TGTTTGAACTTGGTGGGGCT	NM_010442.2
Slc7a11	F: TTACCACCATCAGTGCGGAG<br>R: AACCTGGAGACAGCGAACAC	NM_011990.2
Nrf2	F: AAGAATAAAGTCGCCGCCCA<br>R: AGATACAAGGTGCTGAGCCG	NM_001399226.1
Rpl-19	F: TTTTGCCCGACGAAAGGGTA<br>R: TCCATGAGGATGCGCTTGTT	NM_001159483.1

Supplementary Table S2. Details of antibodies used in the experiment

Antibodies	Source	Catalogue NO.	Dilution
Primary antibody			
GPX4	Bioworld	BS7323	1:1000
PTGS2	Abmart	TA7003	1:1000
SRXN1	Proteintech	14273-1-AP	1:100~1:1000
NRF2	Proteintech	16396-1-AP	1:1000
HO-1	Abclonal	A1346	1:100~1:1000
NQO1	Abclonal	A19586	1:1000
SLC7A11	Abmart	T57046	1:1000
LC3B	Abmart	T55992	1:100~1:1000
Tubulin-α	Bioworld	BS1699	1:10000
Secondary antibody			
Goat anti-Rabbit IgG (H+L)-HRP	Bioworld	BS13278	1:10000
Goat anti-Rabbit IgG (H+L)-Alexa Fluor® 488	Abcam	Ab150077	1:1000

1.Quantitative proteomics analysis

1.1 Protein Preparation and TMT Labelling

HT-22 cells were cultured in 10 cm^2 plates overnight and treated with DMSO or 10 μM erastin for 24 h. Cells were lysed in lysis buffer at 70 Hz for 120 s. The lysed tissue samples were centrifuged, and the supernatant was collected. The protein concentrations were measured by BCA Kit. After quantification, the protein extracted from each sample was digested with 0.5 $\mu g \cdot \mu L^{-1}$ trypsin solution. Peptides in each group were labelled with TMT labels.

1.2 High pH Reversed-Phase Fractionation and LC-MS/MS Analysis

The TMT-labelled peptides were dissolved in 100 μL buffer A (98% double-distilled water, 2% acetonitrile, pH 10) and fractionated via high pH reversed-phase fractionation chromatography with a RIGOL L-3000 HPLC System. Fractions were collected every 1.75 min in 45 tubes and then dried and combined into 10 tubes for further LC-MS/MS analysis. LC-MS/MS analysis was performed with an EASY-nLC 1000 System coupled online to a Q Exactive Mass Spectrometer with an EASY-Spray Ion Source. The samples were loaded onto an Acclaim PepMap 100 precolumn. Peptide separation was conducted using an EASY-Spray column with buffer A (100% ultrapure water and 0.1% formic acid) and buffer B (100% acetonitrile and 0.1% formic acid) at a flow rate of 350 $nL \cdot min^{-1}$. The eluted peptides were analysed using the Q Exactive online system. MS data acquisition was performed using a data-dependent top 20 method.

1.3 Protein Identification and Quantitative Analysis

The LC-MS/MS raw data were searched against the mouse FASTA database from UniProt using Proteome Discoverer version 2.1. The false discovery rate (FDR) was set as <0.01. Detection of at least one unique peptide per protein was set as the requirement for protein identification. The protein quantitative analysis was based on reporter ion peak intensity. Differentially expressed proteins were identified through ratio-fold change as well as P value calculated with a t-test. The up- and downregulation thresholds were set at an average ratio − fold change > 1.2 with a P value < 0.05 and an average ratio − fold change < 1/1.2 with a P value < 0.05; the proteins that satisfied

the set thresholds were collected as “Differentially expressed proteins” for further bioinformatics analysis.

1.4 Bioinformatics Analysis

Functional annotations of differentially expressed proteins were performed through Gene Ontology (GO) enrichment analysis (http://www.geneontology.org/). Proteins were classified by GO annotation into three categories: biological process, cellular compartment and molecular function. For each category, a two-tailed Fisher’s exact test was employed to test the enrichment of the differentially expressed protein against all identified proteins. The GO with a corrected p-value < 0.05 is considered significant. Molecular function describes activities, such as catalytic or binding activities, that occur at the molecular level.
